# DRESS syndrome in the setting of oxacillin therapy—a call for better patient preparedness: a case report

**DOI:** 10.1186/s13256-021-03202-9

**Published:** 2021-12-27

**Authors:** Hawa Ozien Abu, Sajjadh M. J. Ali, Anil Phuyal, Akil Sherif, Gregory T. Williams, Iryna Chastain

**Affiliations:** 1 Department of Internal Medicine, Saint Vincent Hospital, Worcester, MA 01608 USA; 2 Division of Infectious Disease, Reliant Medical Group, Worcester, MA 01608 USA; 3 Hospital Medicine, Reliant Medical Group, Worcester, MA 01608 USA

**Keywords:** Drug reaction with eosinophilia and systemic symptoms (DRESS) syndrome, Oxacillin therapy, Patient-centered care, Patient education, Case report

## Abstract

**Background:**

Drug reaction with eosinophilia and systemic symptoms syndrome is a rare but severe and potentially life-threatening hypersensitivity reaction, with significant morbidity and mortality. The clinical presentation of drug reaction with eosinophilia and systemic symptoms may include extensive skin rash, fever, lymphadenopathy, internal organ involvement, eosinophilia, and atypical lymphocytosis, most commonly due to drug-induced reaction. Our case is a rare occurrence of drug reaction with eosinophilia and systemic symptoms syndrome in the setting of oxacillin therapy.

**Case presentation:**

A 55-year-old Caucasian male presented to the emergency department on account of acute onset, 2-day history of generalized pruritic rash with associated fever, occurring 3 weeks after commencing therapy with intravenous oxacillin for methicillin-sensitive *Staphylococcus aureus* bacteremia. He had no known drug allergies. Two days prior to hospitalization, he had a telehealth visit with the infectious diseases specialist on account of his rash, and was recommended to use oral diphenhydramine. However, with the onset of fever and persistence of his rash, he was advised to discontinue the oxacillin and present to the emergency department. On examination, he was febrile at 101.2 °F and had a generalized blanchable maculopapular and morbilliform rash involving the face, trunk, upper and lower extremities, but sparing the palms, soles, and oral mucosa. He had palpable nontender lymph nodes in the cervical and inguinal regions bilaterally. Laboratory studies revealed atypical lymphocytosis, eosinophilia, neutrophilia, and elevated serum transaminases. He was started on intravenous diphenhydramine and admitted to the in-patient medical service. On the second day of hospitalization, his fever resolved. However, his rash was persistent and generalized, as well as elevated transaminases and an abnormal cell count on the second day of hospitalization. To complete his 6-week course of antibiotics for methicillin-sensitive *Staphylococcus aureus* bacteremia, he was switched to an alternative therapy with cefazolin, and he was scheduled for weekly follow-up assessments following hospital discharge.

**Conclusions:**

Healthcare providers should increasingly be aware of the significant morbidity and mortality attributable to drug reaction with eosinophilia and systemic symptoms syndrome and the potential medications which may incite such life-threatening reactions. Early recognition of drug reaction with eosinophilia and systemic symptoms syndrome and prompt institution of management strategies can promote improved clinical outcomes. Enhanced patient–provider communication strategies should be implemented to better prepare patients for the likelihood of such drug reactions, with the goal of improving patient-centered care and adherence with treatment strategies.

## Introduction

Drug reaction with eosinophilia and systemic symptoms (DRESS) syndrome is a rare hypersensitivity reaction occurring in approximately 1/1000–1/10,000 of drug exposures, and is potentially fatal in up to 10–20% of cases [1, 2]. The clinical manifestations of DRESS syndrome are not immediate and typically appear between 2 and 8 weeks after initiation of the triggering agent [3]. Patients with DRESS syndrome commonly present with rash, fever, lymphadenopathy, and eosinophilia, however, the hallmark of DRESS syndrome is the presence of internal organ dysfunction typically involving the liver, kidneys, heart, or lungs [4].

The pathophysiologic basis of DRESS syndrome is incompletely understood. Hypothesized mechanisms comprise a complex interaction between any of the following: (i) accumulation of drug metabolites due to genetic deficiency of detoxifying enzymes and subsequent drug specific T-cell response with initiation of an inflammatory cascade, activation of eosinophils, and interleukin-5 release; and (ii) a viral–drug interaction commonly observed with human herpes virus-6 (HHV-6), cytomegalovirus (CMV), and Epstein–Barr virus (EBV) [3].

The onset of DRESS syndrome is most commonly attributed to the use of antiepileptic medications, allopurinol, antibiotics including sulfonamides, and vancomycin. Among the list of 50 offending agents implicated in DRESS, oxacillin is not listed [5, 6]. We report a case of oxacillin-induced DRESS syndrome in an adult male patient. Cases of oxacillin induced DRESS syndrome are rarely described in the literature [7, 8], further emphasizing the need for healthcare providers to be increasingly aware of the possibility of DRESS syndrome in the setting of oxacillin use, and to ensure optimal patient education and preparedness for the occurrence of this potentially life-threatening reaction if not promptly detected and managed appropriately.

## Case presentation

**Fig. 1:**
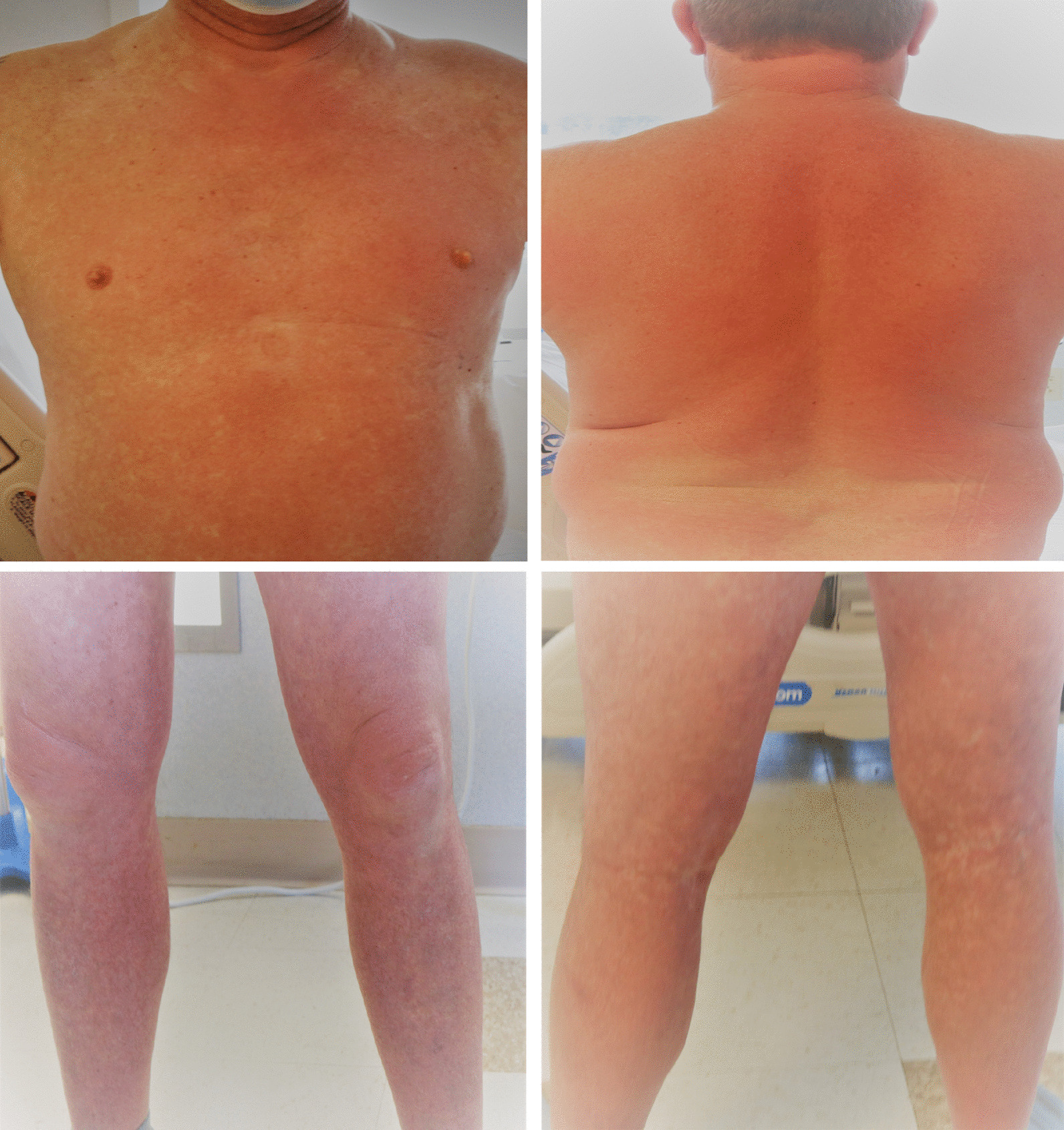
Patient with generalized blanchable maculopapular and morbilliform rash involving: the face and anterior trunk (top left); neck and posterior trunk (top right); anterior thighs, knees and legs (bottom left); and the posterior thighs, popliteal fossa, and calf (bottom right)

A 55-year-old Caucasian male with significant past medical history of hypertension, hyperlipidemia, patent foramen ovale, heart failure with preserved ejection fraction, impaired glucose tolerance, and obesity, presented to the emergency department on account of sudden onset generalized maculopapular rash involving his face, extremities, and trunk, with associated fever and chills. He was hospitalized 1 month earlier and managed for methicillin-sensitive *Staphylococcus aureus* (MSSA) bacteremia and thoracic vertebrae (T11–T12) discitis. During his recent hospitalization, he presented with mid-back pain, fever, and chills. His blood cultures revealed growth of MSSA that was pansensitive to oxacillin, cefazolin, ampicillin/sulbactam, clindamycin, daptomycin, ciprofloxacin, tetracycline, trimethoprim/sulfa, and vancomycin. Magnetic resonance imaging (MRI) of the thoracolumbar spine revealed T11–T12 discitis with no evidence of epidural collection or abscess (Fig. 1). Transthoracic and transesophageal echocardiogram revealed his underlying patent foramen ovale, with no evidence of endocarditis or valvular vegetations. He was reviewed by the infectious disease specialist and managed with intravenous oxacillin 2 g administered every 4 hours during his hospitalization. He had resolution of his fever with intermittent mid-back pain. Repeat blood cultures showed no organism growth. He was discharged on intravenous oxacillin 2 g 4 hourly based on a scheduled 6-week course to be administered via a peripherally inserted central catheter (PICC) line.

The patient remained apparently well until 3 weeks after the start of antibiotic therapy, when he developed sudden onset generalized skin rash with associated pruritus, fever, and chills. He had no recent travel or contact with anyone who had a similar rash. He was sexually active in a monogamous heterosexual relationship. He had no associated symptoms of runny nose, sore throat, cough, shortness of breath, joint pain, or myalgia. He had intermittent back pain on account of thoracic discitis, with no localized swelling or limited mobility of his spine. He was up to date on his vaccinations. His home medications included lisinopril, probiotics, ibuprofen, and a remaining 3-week course of oxacillin. He had no known drug allergies. He had no known history of direct exposure to environmental pollutants. He had 30 pack year history of smoking cigarettes. He quit smoking 15 years prior to the current hospitalization. He consumed two standard drinks of alcohol per week. He had no history of recreational drug use. He worked as a truck driver transporting gasoline and diesel, and used personal protective equipment including respirator masks and gloves. He had significant history of asthma in his brother. No other known history of hypersensitivity in his family.

**Fig. 2:**
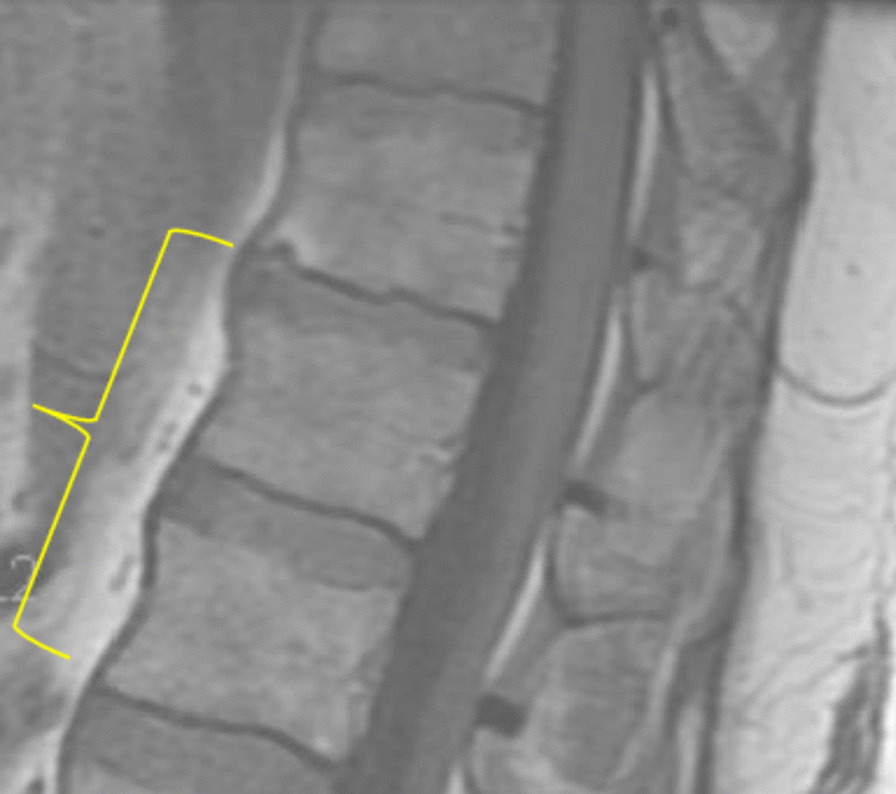
Thoracolumbar spine MRI during the patient’s previous hospitalization, which showed mildly enhancing central T11–T12 intervertebral disc with adjacent endplate enhancement suggestive of early discitis. No discrete epidural collection or abscess identified

Due to the sudden and unexpected onset of generalized rash, he was apprehensive and immediately set up a telehealth visit with his infectious diseases specialist who recommended the use of over-the-counter oral diphenhydramine. However, with the onset of fever and persistence of his rash, he was advised to discontinue the use of oxacillin and present to the emergency department. On arrival, he was alert, oriented to person, place, and time, and not in acute distress. He had unstable vital signs including fever at 101.2 °F, elevated blood pressure 152/87 mmHg, tachycardia with heart rate 136 beats per minute, respiratory rate 18 breaths per minute, and pulse oximetry 96% on room air. He had generalized blanchable maculopapular and morbilliform rash involving the face, trunk, upper and lower extremities, and sparing the palms, soles, and oral mucosa (Fig. 2). He had palpable nontender lymph nodes in the cervical and inguinal regions bilaterally. His conjunctiva was not pale and sclera anicteric. He had clear vesicular breath sounds bilaterally. He had rapid and regular heart rate, normal rhythm, and normal heart sounds with no murmurs. His abdomen was obese, nontender, no palpable masses or hepatosplenomegaly, with the presence of normoactive bowel sounds. His neurological examination revealed normal cranial nerves II–XII with no neurologic deficits, 5/5 motor strength in his upper and lower extremities, normal deep tendon reflexes, strength, and sensation bilaterally. No swelling or tenderness on his spine. He had a normal gait and balance, with no evidence of cerebellar dysfunction. Hematologic lab values revealed white blood cell count 4.6 × 1000/µL (reference range 4–11 × 1000/µL), atypical lymphocytosis 0.5 × 1000/µL; 13% (reference range 0–4.5 × 1000/µL; 0–7%), eosinophilia 0.6 × 1000/µL (reference range 0–0.4 × 1000/µL), neutrophilic bandemia 18% [reference range 0–8%]. He had elevated transaminases including aspartate transaminase (AST) 101 U/L (reference range 0–55 U/L), alanine transaminase (ALT) 115 U/L (reference range 0–44 U/L), and alkaline phosphatase (ALP) 216 U/L (reference range 25–150 U/L). Inflammatory markers were elevated, including erythrocyte sedimentation rate (ESR) 31 20 mm/hour (reference < 20 mm/hour) and C-reactive protein (CRP) 34.5 mg/dL (reference < 8 mg/dL). He was further evaluated to rule out other potential infectious etiology, which were unrevealing as urine, blood, and PICC line catheter tip cultures showed no growth of organisms. Blood culture samples for aerobic and anerobic bacteria were obtained by venipuncture using two sets of sterile blood culture bottles and cultured in BacT-ALERT. No organism growth was seen in the blood cultures after 5 days of incubation. No fungal organisms were detected. Tests for herpes simplex virus (HSV) I and II immunoglobulin (IgM) antibody, infectious mononucleosis, rubeola IgM, urine legionella antigen, hepatitis A, B, and C panel, and CMV DNA polymerase chain reaction and IgM antibody were all negative, as well as human immunodeficiency virus (HIV) 1 and 2 antigen/antibody fourth generation test, which was nonreactive, and corona virus 2019 (COVID-19) SARS-CoV-2 was undetected (Table 1).

**Table 1:** Laboratory results of hematologic, biochemical, serological, and microbiological investigations during the patient’s current hospitalization and at follow-up

Laboratory tests (reference range and units)	Hospital Day 1	Hospital Day 2	Hospital Day 3	Follow-up Week 1	Follow-up Week 2
Complete blood count					
White blood cell count (3.9–11.0 × 1000/µL)	4.6	4.0	4.2	6.9	7.6
Red blood cell count (4.30–5.80 million/µL)	4.39	4.61	4.14	4.22	4.31
Hemoglobin (12.5–17.0 g/dL)	13.3	13.9	12.3	12.5	13.1
Hematocrit (36.0–50.0%)	37.8	39.4	35.5	36.7	38.2
Mean corpuscular volume (80–100 fL)	86	86	86	87.0	88.6
Mean corpuscular hemoglobin (27–33 pg)	30	30	30	29.6	30.4
Mean corpuscular hemoglobin concentration (31–36 g/dL)	35	35	35	34.1	34.3
Red cell distribution width (11.4–14.4%)	13.7	13.9	14.0	14.0	13.9
Platelet count (150–450 × 1000/µL)	201	193	195	296	463
Mean platelet volume (7.0–11.0 fL)	10.3	10.2		10.6	10.5
Reticulocyte count (1.6–2.6 %)		1.6			
Absolute reticulocyte (35–101 billion/L)		74.7			
Neutrophil count (1.8–7.0 × 1000/µL)	3.4			4.0	4.8
Segmented neutrophils (40–75%)	73	24		58.2	63.4
Segmented neutrophils count (1.8–7.0 × 1000/µL)		1.0			
Band neutrophils (0.0–8.0%)		18			
Band neutrophil count (0.0–0.7 × 1000/µL)		0.7			
Lymphocytes (%)	13	18		21.4	23.3
Lymphocyte count (0.7–4.5 × 1000/µL)	0.6	0.7		1.5	1.8
Atypical lymphocyte count (0.0–0.45 × 1000/µL)		0.5			
Atypical lymphocytes (0.0–7.0%)		13			
Monocytes (%)	7	11			
Monocyte count (0.1–0.8 × 1000/µL)	0.3	0.4		0.4	0.6
Eosinophils (%)	7	15			
Eosinophil count (0.0–0.4 × 1000/µL)	0.3	0.6		0.89	0.25
Basophils (%)	0	1.0		1.0	
Basophil count (0.0–0.2 × 1000/µL)	0.0	0.0		0.07	0.16
Metamyelocytes (%)		0			
Myelocytes (%)		0			
Promyelocytes (%)		0			
Blast cells (%)		0			
Inflammatory markers					
Erythrocyte sedimentation rate westergren (<20 mm/hour)	31.0			36.0	29.0
C-reactive protein (< 8.0 mg/dL)	34.5			17.3	7.7
Haptoglobin (29–370 mg/dL)	252				
Basic metabolic panel					
Sodium (135–146 mmol/L)	135	137	139		140
Potassium (3.5–5.3 mmol/L)	3.9	4.4	4.0		4.5
Chloride (98–110 mmol/L)	101	102	100		104
Carbon dioxide (20–32 mmol/L)	22	25	24		24
Blood urea nitrogen (7–25 mg/dL)	14	12	12		8
Creatinine (0.70–1.33 mg/dL)	1.29	1.11	1.23		0.94
Estimated GFR (≥ 60 mL/minute/1.73 m^2^)	62.0	74.4	65.7		91
Mean blood glucose (65–99 mg/dL)		140	130		118
Calcium (8.6–10.3 mg/dL)	9.2	9.5	8.9		9.5
Lactic acid (0.5–2.0 mmol/L)	1.0	1.4			
Hepatic function panel					
Total bilirubin (0.1–1.2 mg/dL)	0.8	0.7	0.5		
Direct bilirubin (0.0–0.4 mg/dL)		0.3			
Aspartate transaminase (10–35 U/L)	101	82	46	29	12
Alanine transaminase (9–46 U/L)	115	128	91	37	6
Total alkaline phosphatase (25–150 U/L)	216	230	199		
Total protein (6.0–8.5 g/dL)	6.8	7.3	6.4		
Albumin (3.5–5.5 g/dL)	3.8	4.0	3.6		
Coagulation profile					
PT (9.1–12.0 seconds)		10.6			
INR (2.0–3.5)		1.0			
APTT (25.0–35.0 seconds)		27.1			
Enzyme levels					
Lactate dehydrogenase (< 226 U/L)		414			
Creatine kinase (24–204 U/L)		20			
Troponin T (< 0.030 ng/mL)		<0.030			
Amylase (31–124 U/L)		36			
Lipase (0–59 U//L)		43			
Serology					
Coronavirus-19 SARS COV2 (PCR)		Not detected			
RSV nasal swab		Negative			
CMV IgM Ab			< 30.0		
CMV DNA Quant PCR			Negative		
Hepatitis A IgM Ab			Negative		
Hepatitis B surface Ag			Negative		
Hepatitis B surface Ab			Nonreactive		
Hepatitis B core IgM Ab			Negative		
Hepatitis C antibody			< 0.1		
HSV I and II IgM Ab			< 0.91		
Human herpes virus type 6, IgM			< 1:10 [Negative]		
Human herpes virus type 6, IgG			0.96 (Equivocal)		
HIV 1 and 2 Ag/Ab, 4th Gen			Nonreactive		
Infectious mononucleosis assay			Negative		
Influenza type A Ag		Negative			
Influenza type B Ag		Negative			
Rubeola (measles) IgM			< 0.91		
Aerobic and anaerobic blood cultures			No organism growth		
Fungal blood cultures			No organism growth		
Peripherally inserted central catheter tip culture			No organism growth		
Urinalysis					
Urine color		Yellow			
Urine appearance		Clear			
Urine pH		6.0			
Urine specific gravity		1.015			
Urine protein		Negative			
Urine ketones		Negative			
Urine blood		Negative			
Urine nitrite		Negative			
Urine bilirubin		Negative			
Urine urobilinogen		1.0			
Urine leukocyte esterase		Negative			
Urine sediment examination			Rare WBC Few bacteria		
Urine eosinophilic smear			None seen		
Urine culture indicated?		No			
Urine glucose		Negative			

*Ab* antibody, *Ag* antigen, *APTT* activated partial thromboplastin time, *CMV* cytomegalovirus, *GFR* glomerular filtration rate, *HIV* human immunodeficiency virus, *HHV-6* human herpes virus-6, *HSV* herpes simplex virus, *INR* international normalized ratio, *PCR* polymerase chain reaction, *PT* prothrombin time, *RSV* respiratory syncytial virus, *WBC* white blood cell

He was managed as a case of DRESS syndrome with intravenous fluids (2 L of 0.9% normal saline), intravenous diphenhydramine HCL 25 mg 8 hourly as needed for itching (three doses), acetaminophen 650 mg 8 hourly via oral route as needed for fever (three doses), and topical application of calamine lotion three times daily. For deep venous thrombosis prophylaxis, he received subcutaneous heparin sodium 5000 units every 8 hours (four doses), for prevention of stress ulcers he received pantoprazole 40 mg (two doses), and two doses of *Lactobacillus acidophilus* tablet. His home medication of lisinopril 10 mg daily for hypertension was withheld for concerns for potential acute kidney injury. On the second day of hospitalization, there was resolution of his fever. However, his generalized rash persisted, alongside elevated transaminases, and abnormal cell count. He remained hemodynamically stable and did not develop any new symptoms. To complete his course of antibiotic therapy for MSSA bacteremia, he was switched to an alternative therapy with cefazolin 2 g every 8 hours for 6 weeks. He received four doses of cefazolin prior to hospital discharge and was closely monitored to ensure that he had no reaction to cefazolin. The choice of cefazolin was based on the considerable severity of his thoracic discitis and the intention to appropriately manage him with the most sensitive antibiotic available. The possibility of cross reaction with cefazolin was considered less likely and he was closely monitored for any adverse reaction with weekly follow-up assessments in the outpatient setting. He tolerated the cefazolin without any untoward reaction and successfully completed his course of treatment.

Within a week of follow-up with the infectious disease specialist, he had notable clinical improvement with resolution of his rash and normalization of his liver enzymes. However, he had persisting mild anemia (hemoglobin 12.5 g/dL), eosinophilia (eosinophils 890 cells/µL), and elevated inflammatory markers including ESR (36 mm/hour) and CRP (17.3 mg/dL). In the subsequent week of follow-up, there was no recurrence of his rash, his liver enzymes were not elevated, he had improving anemia with hemoglobin at 13.1 g/dL, and resolving inflammatory markers with ESR at 29 mm/hour and CRP within normal limits. No new medication was initiated during outpatient follow-up with the infectious disease specialist. Upon resolution of his symptoms and completion of his antibiotic therapy, he reported improvement in his quality of life and intention to return to work. He was medically cleared and deemed fit to work. The patient was counseled by the infectious disease specialist on potential triggers of DRESS syndrome and avoiding future occurrence.

## Discussion

Making a diagnosis of DRESS syndrome can be challenging but should be approached systematically based on the patient’s clinical presentation, consideration of the latent period between initiation of a new high-risk medication and development of symptoms, as well as excluding other non drug-induced conditions [4]. Multiple diagnostic criteria have been developed to promote a more standardized approach to the diagnosis and management of DRESS syndrome. Among hospitalized patients with drug-induced rash, the Registry of Severe Cutaneous Adverse Reaction (RegiSCAR) group has suggested the criteria for making a diagnosis of DRESS syndrome [9]. While these criteria were originally developed as a tool for retrospective validation of suspected cases, they are frequently used to support a clinical diagnosis of DRESS syndrome. However, many characteristic features of this condition may not be present concomitantly given its dynamic nature. Therefore, a high degree of suspicion becomes essential when entertaining a diagnosis of DRESS syndrome.

In this case report, we present a 55-year-old adult male who developed generalized pruritic rash and fever 3 weeks after commencing therapy with intravenous oxacillin for MSSA bacteremia. Based on the validated RegiSCAR classification of DRESS (classified as either possible, probable, or definite) [9], our patient had a score of 6, which is equivalent to a definite classification of DRESS based on the presence of fever > 38 °C, > 50% involvement of generalized skin rash, atypical lymphocytosis, eosinophilia, liver involvement characterized by elevated transaminases, enlarged lymph nodes at two sites (cervical and inguinal), and at least three biological tests including HIV, HHV-6, CMV, EBV, which were negative to exclude other differential diagnosis. Furthermore, the period of onset of symptoms in DRESS is approximately 2–8 weeks, with an average of 3 weeks [3]. In our patient, the onset of rash and fever was within 3 weeks of exposure to oxacillin, which falls within the commonly reported period of the onset of DRESS syndrome after exposure to a trigger agent.

From review of prior literature, there is only one other reported case of DRESS syndrome associated with oxacillin use in the adult population, however, this is the first case report of oxacillin-related DRESS syndrome with significant internal organ involvement occurring in an adult male. The previous case, reported in 2019, described a 52-year-old male managed for an epidural abscess secondary to oxacillin-sensitive *Staphylococcus aureus*, who was initiated on an extended course of oxacillin and rifampin, and on day 22 of treatment, developed new-onset fever, rash, and agranulocytosis, with resolution of his leukopenia after discontinuing oxacillin therapy [7]. The referenced case report differs from the present case as there was no involvement of the patient’s internal organs, while our patient had a more complicated hospital course with new-onset transaminitis requiring close monitoring and follow-up while hospitalized and at discharge. Our case further emphasizes the need for healthcare providers to promptly identify DRESS syndrome as there may be life-threatening organ involvement requiring urgent treatment strategies.

There are currently no established guidelines in the management of DRESS syndrome. Prompt recognition of the onset of DRESS syndrome and removal of the inciting agent is a key strategy to limiting further internal organ damage. Controversies still exist regarding the use of steroid therapy. The French Society of Dermatology in 2010, outlined guidelines on the therapeutic approach to managing DRESS syndrome [10]. They recommend the use of systemic corticosteroids in patients with signs of severe features of DRESS including transaminases greater than five times normal, renal involvement, lungs, or cardiac involvement. Furthermore, they propose the use of steroids in combination with ganciclovir in patients with signs of severity and confirmation of a major viral reactivation of HHV-6. Isolated transaminitis is the most common laboratory evidence of hepatitis in DRESS syndrome [11]. In more severe cases, progression to fulminant hepatic failure may occur in as many as 1 in 10 patients, and this is a major recognized cause of mortality among those with DRESS syndrome [1]. Although steroids are a mainstay in the treatment of DRESS, they are primarily indicated only in severe cases with life-threatening organ involvement and cases requiring intensive care unit (ICU) stabilization. Mild cases with no organic involvement or only mild involvement of the liver usually does not warrant steroid therapy. This is especially relevant in the setting of sepsis or other active ongoing infection where steroid therapy can pose a significant risk in terms of worsening sepsis. There is also evidence to suggest that the resulting immunosuppression from steroid therapy may cause reactivation of viruses, such as HHV-6 and CMV [12].

In our patient, the extent of derangement of transaminases was not excessive and the transaminitis plateaued early during the hospitalization. Taking into consideration his history of recent bacteremia with discitis, systemic steroids were not instituted in his management as the risk of steroid therapy would have outweighed the potential benefits. In addition, given that his HHV-6 immunoglobulin G titer did not confirm viral reactivation, ganciclovir was not indicated in his management.

We recommend a multidisciplinary and team-based approach in emphasizing to patients the typical symptoms of DRESS and the timeframe for its onset after initiating high-risk medications. Patient–provider communication can be enhanced by having multiple healthcare providers emphasize to patients the time interval most concerning for the onset of DRESS syndrome. For example, the clinician prescribing a potentially high-risk medication can inform the patient of the period to be vigilant for any possible onset of fever and rash. Furthermore, the pharmacist dispensing the medication can further emphasize to the patient the time interval most concerning for the development of DRESS syndrome. In addition, a medication alert system can be designed, by which patients can check in weekly into an electronic system that would inquire about possible development of skin rash, fever, or other concerning features that may arise from certain high-risk medications. This multidisciplinary approach is of utmost clinical importance as it not only prepares patients for possible onset of DRESS syndrome but would also facilitate its prompt identification and potentially reduce fatal complications that may be associated with delayed recognition and management. However, engaging patients in this regard should not be conducted in a manner that could instigate apprehension and over vigilance, but rather in a fashion that would enhance medication adherence and early presentation with the onset of untoward symptoms, and potentially reduce life-threatening complications.

Key strategies employed in managing our patient include prompt discontinuation of oxacillin, avoiding steroid therapy due to potential risks, switching to alternative antimicrobial therapy for his MSSA bacteremia, and supportive management of his symptoms in a multidisciplinary manner that facilitated patient centered care with closer monitoring and follow-up.

## Conclusion

Given the significant morbidity and mortality associated with DRESS syndrome, healthcare providers should be increasingly aware of this severe and potentially life-threatening hypersensitivity reaction when prescribing high-risk medications associated with this condition. The diagnosis of DRESS can be facilitated by criteria such as the RegiSCAR, but a high degree of suspicion must be entertained in atypical cases as diagnostic criteria may not occur concurrently. Of key importance in the management of DRESS syndrome is promptly withdrawing the offending agent. Additionally, steroids may be withheld in mild cases, especially in the setting of active infection. Furthermore, through a multidisciplinary approach, a high level of patient engagement could be fostered to better prepare patients and their caregivers for the possible onset of hypersensitivity reaction, prompt recognition of symptoms, removal of the inciting trigger, and avoiding fatal complications associated with DRESS syndrome.

## Data Availability

Data sharing not applicable to this article as no datasets were generated or analyzed during the current study.

## Abbreviations

ALP: Alkaline phosphatase
ALT: Alanine transaminase
APTT: Activated partial thromboplastin time
AST: Aspartate transaminase
CMV: Cytomegalovirus
COVID-19: Coronavirus disease 2019
CRP: C-reactive protein
DRESS: Drug reaction with eosinophilia and systemic symptoms
EBV: Epstein–Barr virus
ESR: Erythrocyte sedimentation rate
GFR: Glomerular filtration rate
HIV: Human immunodeficiency virus
HHV-6: Human herpes virus-6
HSV: Herpes simplex virus
INR: International normalized ratio
MSSA: Methicillin sensitive *Staphylococcus aureus*
PCR: Polymerase chain reaction
PICC: Peripherally inserted central catheter
PT: Prothrombin time
RegiScar: The Registry of Severe Cutaneous Adverse Reaction
T11–T12: Thoracic vertebrae
